# Cellular and Molecular Mechanisms of Oxidative Stress in Wound Healing

**DOI:** 10.1155/2022/9785094

**Published:** 2022-06-17

**Authors:** Reggiani Vilela Gonçalves, Mariella Bontempo Freitas, Debora Esposito

**Affiliations:** ^1^Animal Biology Department, Federal University of Viçosa, Viçosa, 36570-000 Minas Gerais, Brazil; ^2^Animal Science Department, Regenerative Medicine, North Carolina State University, 28081 North Carolina, USA

Wound healing is a complex biological process characterized by cell activation and by the release of many growth factors responsible to restore tissue integrity after injury [1]. This process can be divided into three stages: inflammation, proliferation, and remodeling [2]. At the beginning of the process, the collagen exposed initiates the coagulation cascade forming the platelet cap, and as the process progresses occur, the release of many cytokines and growth factors promotes cell and vascular proliferation [3] The next stage is the proliferative phase, characterized by the synthesis of the granulation tissue rich in vessels, cells, and type III collagen. This tissue serves as a framework for type I collagen deposition responsible for the force and resistance to the tissue characterizing the third stage known as remodeling [4].

In skin lesions, the synthesis of free radicals and reactive oxidative species (ROS) by inflammatory cells contributes to the defense against pathogens and mediates important intracellular pathways for the resolution of the inflammatory phase [5]. However, excessive amounts of free radicals and ROS promote tissue oxidative stress, causing deleterious effects on cell membranes, proteins, and nucleic acids [5]. Generally, the higher production of oxidative compounds occurs in the wound healing process associated with diseases such as metabolic (i.e., diabetes mellitus and vasculopathy) and microbial diseases (i.e., bacterial, fungal, and parasitic infections) [6]. So, we believed that understanding the relationship between the interaction of these different mechanisms and the oxidative process is important to provide a guideline for decision-makers in the choice of better treatments and consequently accelerate skin wound closure.

Thinking in the relationship among different phases of the wound healing process, different treatments, and redox metabolism, this special issue gathered 9 studies using in vivo and in vitro analyses to understand the cellular, immunological, and morphological bases associated with the recovery process of the injured tissue. Concerning new therapies, some studies have shown that external agents such as peptides obtained from animals, plant extract, and acidic electrolyzed water and antibiotics were considered as a rich source of active molecules with potential relevance and applicability indirect or complementary strategies focused on tissue repair. These results showed us that several compounds with proliferation and migration cell potential, antimicrobial properties, and inflammatory modulation have been used with a success in regenerative medicine. However, there is still a gap in knowledge about the main pathways involved in the healing process, and interestingly, some papers published in this edition analyzed the relationship between cell pathway signaling and immunoregulatory genes and wound healing trying to fill the gaps that still exist in this area.

In another study from this issue, the authors showed that mouse tendon-derived cells were highly tolerant to hypoxic environments. During hypoxia/reoxygenation (HR), the authors observed a concentration-dependent increase in cell viability. Under H/R conditions, the expression of vascular endothelial growth factor-A (VEGF-A) and hypoxia-inducible factor HIF-1*α* was opposite, whereas type I and type III collagen was downregulated. Considering that although oxidative stress was induced after a period of ischemia, stress responses did not affect cell morphology and growth.

In this issue, in vitro studies have contributed to the field of cellular and molecular mechanisms of oxidative stress in wound healing. One study has shown that medicine dressings made from genetically modified flax fibers had a stronger impact on fibroblasts' proliferative activity, keratinocytes, and microvascular endothelium than traditional flax fiber in several cell lines, such as fibroblasts, epidermal keratinocytes, endothelial, and carcinoma cells. Free oxygen radical levels were reduced in both traditional and genetically modified fibers, corroborating the classical use of this plant as a dressing. Reduced reactive oxygen species production is associated with the protection of DNA damage and favors wound healing. However, especially in difficult-to-heal wounds, other mechanisms such as those which were higher in genetically modified flax fibers wound will be critical to a successful treatment.

We sincerely hope that this special issue's readers will find these findings interesting, advancing knowledge on molecular mechanisms associated with oxidative stress and wound healing.
